# Myocardial proteomic profile in pulmonary arterial hypertension

**DOI:** 10.1038/s41598-020-71264-8

**Published:** 2020-09-01

**Authors:** Mateusz K. Hołda, Aneta Stachowicz, Maciej Suski, Dorota Wojtysiak, Natalia Sowińska, Zbigniew Arent, Natalia Palka, Piotr Podolec, Grzegorz Kopeć

**Affiliations:** 1 HEART - Heart Embryology and Anatomy Research Team, Department of Anatomy, Jagiellonian University Medical College, Kopernika 12, 31-034 Kraków, Poland; 2 Department of Cardiac and Vascular Diseases, Jagiellonian University Medical College, Kraków, Poland; 3 Division of Cardiovascular Sciences, The University of Manchester, Manchester, UK; 4 Department of Pharmacology, Jagiellonian University Medical College, Kraków, Poland; 5 Department of Animal Genetics, Breeding and Ethology, University of Agriculture in Cracow, Kraków, Poland; 6 Center of Experimental and Innovative Medicine, University Center of Veterinary Medicine JU-AU, University of Agriculture in Cracow, Kraków, Poland

**Keywords:** Cardiovascular biology, Anatomy

## Abstract

Pulmonary arterial hypertension (PAH) is a rare, fatal, and incurable disorder. Although advances in the understanding of the PAH pathobiology have been seen in recent years, molecular processes underlying heart remodelling over the course of PAH are still insufficiently understood. Therefore, the aim of this study was to investigate myocardial proteomic profile of rats at different stages of monocrotaline-induced PAH. Samples of left and right ventricle (LV and RV) free wall collected from 32 Wistar rats were subjected to proteomic analysis using an isobaric tag for relative quantitation method. Hemodynamic parameters indicated development of mild elevation of pulmonary artery pressure in the early PAH group (27.00 ± 4.93 mmHg) and severe elevation in the end-stage PAH group (50.50 ± 11.56 mmHg). In early PAH LV myocardium proteins that may be linked to an increase in inflammatory response, apoptosis, glycolytic process and decrease in myocardial structural proteins were differentially expressed compared to controls. During end-stage PAH an increase in proteins associated with apoptosis, fibrosis and cardiomyocyte Ca^2+^ currents as well as decrease in myocardial structural proteins were observed in LV. In RV during early PAH, especially proteins associated with myocardial structural components and fatty acid beta-oxidation pathway were upregulated. During end-stage PAH significant changes in RV proteins abundance related to the increased myocardial structural components, intensified fibrosis and glycolytic processes as well as decreased proteins related to cardiomyocyte Ca^2+^ currents were observed. At both PAH stages changes in RV proteins linked to apoptosis inhibition were observed. In conclusion, we identified changes of the levels of several proteins and thus of the metabolic pathways linked to the early and late remodelling of the left and right ventricle over the course of monocrotaline-induced PAH to delineate potential therapeutic targets for the treatment of this severe disease.

## Introduction

The pulmonary arterial hypertension (PAH) is characterized by increased vascular resistance in pulmonary arterial circulation. PAH is a rare, fatal, and incurable disorder with an increasing prevalence over time that is estimated to range from 30 to 50 cases per million^1–3^. Chronically elevated blood pressure in the pulmonary arteries activates a right ventricular (RV) adaptive response to the increased afterload. Further decompensation of the adaptive response leads to the development of pressure-overload-induced RV failure^4^. It has been well documented that PAH-related morphological changes not only occur in the RV but in all heart cavities, including the left ventricle (LV)^5,6^.

Apart from RV hypertrophy (increased myocardial mass, thickening of the ventricle wall, and dilatation of the ventricular cavity), significant, but opposite changes occur in the LV at the end stages of the disease: (1) decrease in myocardial mass and (2) reduction in wall thickness and stricture of the ventricle cavity^5,6^. Moreover, hemodynamic disturbances, such as an increase in the RV and decrease in the LV systolic pressure are observed^7^.

Although advances in the understanding of the pathobiology of PAH have been seen in recent years, molecular processes underlying heart remodeling over the course of PAH are still insufficiently understood^8,9^. In particular, there is still incomplete knowledge regarding the mechanisms of LV mass loss and dysfunction, which was completely avoided by researchers until recently^10,11^. Therefore, to enrich our knowledge on this subject, we aimed to assess global quantitative and qualitative protein profile changes in the LV and RV myocardia from rats over the course of monocrotaline-induced PAH. Such a throughput approach of this study may contribute to further understanding of related changes in PAH and facilitate the development of therapeutic targets.

## Material and methods

### Animal model

This study was approved by the 2nd Local Ethical Committee in Cracow, Poland (No 60/2016) and was performed in accordance to the guidelines from Directive 2010/63/EU of the European Parliament on the protection of animals used for scientific purposes. After a two-week quarantine period, on day 0, 66 Wistar male rats (eight weeks old; provided by Experimental Medicine Center of the Medical University of Bialystok, Poland) were randomly assigned to two groups: (1) In the study group, animals (n = 48) were injected intraperitoneally with a single dose of 60 mg/kg monocrotaline in Dulbecco’s phosphate-buffered saline (PBS) (3 mL/kg, Sigma-Aldrich, Germany) medium to induce PAH^12^ and (2) In the control group, rats (n = 18) were injected with the same amount (3 ml/kg) of the medium without drug. Rats were maintained under standard conditions and were fed a normal rat diet.

### Echocardiographic examination

In order to assess the development of PAH and morphometric cardiac parameters, animals in both groups were subject to regular transthoracic echocardiographic (TTE) examinations (Mindray M7 with P12-4s, 4.2–11 MHz transducer, Mindray Bio-Medical Electronics Co., Shenzhen China) performed with blinding on day 0 (prior to intraperitoneal injection) and on days + 5, + 10, + 15, + 20, + 24 and then every three days and on the day of rat euthanasia. The TTE was performed on a conscious animal (without any drug administration) immobilized manually in a supine position on the dorsum. To ensure cooperation of the animals, rats were subjected to extensive handling. Specifically, heart rate, end-diastolic RV free wall thickness (RVFWTd), tricuspid annular plane systolic excursion (TAPSE), and pulmonary artery acceleration time normalized to cycle length (PAAT/CL) were measured in the standard way (at a 10.0 MHz frequency and a rate of 114 frames/sec)^13,14^.

### Experiment's structure

The project evaluated two main endpoints:

1. Early signs of PAH. Point 1 criterion: first morphological lesions of the RV visible on the TTE of rats (RVFWTd > 0.7 mm)^14^. A total of 12 animals from the study group that met this criterion and eight time-paired rats from control group were sacrificed.
2. Heart failure secondary to PAH (end-stage PAH). Point 2 criterion: clinical signs of RV insufficiency up to end-stage circulatory and respiratory insufficiency. A total of 18 animals with heart failure and eight time-paired rats from the control group were sacrificed.

The remaining rats in the study group that have not met endpoint 2 criterion at the assumed experiment time did not develop PAH, and/or died under uncontrolled conditions. Finally remaining two rats from the control group were excluded from the study.

### Hemodynamic examination

On the day of sacrifice, animals were subject to invasive hemodynamic testing. Rats were premedicated and anesthetized with isoflurane. Animals were mechanically ventilated during the whole procedure using a pressure-controlled respirator and a mixture of air and oxygen. Lidocaine (20 mg/ml, B. Braun Melsungen AG, Germany) was used for local infiltration of the surgical sites. Chest cavities were opened via left and right mini thoracotomy at the sixth intercostal space. Heparinized 21G venous cannula were then connected to a pressure recording system (Siemens SC 7,000, Erlangen, Germany) through a saline-filled system that was introduced to the RV and LV via their apexes in order to measure systolic and diastolic blood pressures^15^. The pressure transducer was fixed to the operating table and set at the level of the animal’s heart. The values were registered from 300-s periods of stable signal and means were calculated as output values. Animals were sacrificed after the procedure.

### Animal euthanasia and dissection

Rat sacrifice was performed through overdosing sodium pentobarbital via intraperitoneal administration. Directly after declaring termination of vital functions, the chest cavity was opened. The descending aorta and inferior vena cava were cannulated, blood was removed, and infusion of the body using large volumes of Ringer’s solution (Fresenius Kabi, Germany) was conducted in order to clean the protein material originating from the vascular bed away from the myocardium. Next, the heart and its main vessels were dissected, blot dried, and weighed. Using a stereoscopic microscope, the muscle tissue of the LV and RV free wall and interventricular septum were completely separated from each other and remaining heart structures and then weighed. Tissue samples were divided into adequately large sections and immediately frozen at –80 °C or fixed in 10% buffered paraformaldehyde solution.

### Histological analysis

In order to assess microscopic structure of the myocardium and signs of inflammation histological processing was performed on paraformaldehyde-fixed samples. Briefly, samples were dehydrated in a series of alcohols, cleared in xylene, and embedded in paraffin blocks. Samples were cut into 6-µm sections (Leica RM2146 microtome, Germany) and stained with hematoxylin and eosin (Sigma-Aldrich, Germany). Inflammatory cell infiltration was assessed semi-quantitatively (0 = lack, 1 = low, 2 = moderate, 3 = high, 4 = severe) in the light microscope (Nikon E600, Japan). It has been proven that monocrotaline, apart from its pneumotoxic effects responsible for PAH induction, also presents direct cardiotoxic effects as expressed by myocarditis^16^. In this study, only samples with lower than moderate signs of myocarditis were accepted for further proteomic analysis.

Moreover, 6 μm paraffin sections were cut and placed onto SuperFrost Plus slides (Menzel, Germany). Using Wheat Germ Agglutinin–Alexa Fluor 488 (Invitrogen, USA) and DAPI (4,6-diamidino-2-phenylindole hydrochloride, Invitrogen, USA) sections were stained in a Coplin jar utilizing the protocol described by Bensley et al.^17^. Sections were mounted using ProLong Gold (Invitrogen, USA) and examined with a Zeiss Axio Vision A.2 (Oberkochen, Germany) fluorescence microscope to detect cardiac fibrosis^18^.

### Sample preparation for proteomic analysis

Frozen samples of LV and RV free wall collected from 32 non-inflammatory animals were subject to proteomic analysis: (1) Group I (study group): n = 16 (endpoint 1, early PAH, n = 8; endpoint 2, end-stage PAH, n = 8) and (2) Group II (control group): n = 16 (endpoint 1: n = 8; endpoint 2: n = 8). Each sample was homogenized using a Tissue Lyser LT (Qiagen, Germany) and lysed in a buffer containing 0.1 M Tris–HCl, pH 8.0, 2% sodium dodecyl sulfate, and 50 mM dithiothreitol (Sigma Aldrich, USA) at 96 °C for 10 min. Protein concentration was measured by Pierce 660 nm Protein Assay Kit (Thermo Scientific, USA). Each two samples from one group were pooled and then processed further. Seventy micrograms of protein content were digested using the multiple enzyme digestion filter aided by a sample preparation method (MED FASP)^19,20^ with two enzymes: (1) endoproteinase LysC and (2) trypsin. Next, samples were purified with C18 MacroSpin Columns (Harvard Apparatus, USA) and prepared as recommended by the iTRAQ protocol (ABSciex, USA). Four samples from each group were labeled with iTRAQ reagents as follows: (1) LV in endpoint 1: 113, 115, 117, 119; (2) control to LV in endpoint 1: 114, 116, 118, 121; (3) LV in endpoint 2: 114, 116, 118, 121; (4) control to LV in endpoint 2: 113, 115, 117, 119; (5) RV in endpoint 1: 113, 115, 117, 119; (6) control to RV in endpoint 1: 114, 116, 118, 121; (7) RV in endpoint 2: 114, 116, 118, 121; and (8) control to RV in endpoint 2: 113, 115, 117, 119. Then each group of samples was combined with their respective controls, dried in a vacuum concentrator (Eppendorf, Germany), and dissolved in 0.1% trifluoroacetic acid to purify it with C18 MacroSpin columns (Harvard Apparatus, USA). Eluates were reconstituted in 0.2 ammonium formate, pH 10.0, and subject to fractionation under high pH conditions (Harvard Apparatus, USA). Peptides were eluted in 10 consecutive salt steps (15%, 17.5%, 20%, 22.5%, 25%, 27.5%, 30%, 32.5%, 35%, and 50% acetonitrile in 0.05 M ammonium formate) and dried in a vacuum concentrator.

### LC–MS analysis

Samples were dissolved in 5% acetonitrile with 0.1% formic acid and concentrated on a trap column (Acclaim PepMap100 RP C18 75 µm i.d. × 2 cm column, Thermo Scientific Dionex, USA) and then injected on-line onto a PepMap100 RP C18 75 µm i.d. × 15 cm column (Thermo Scientific Dionex, USA). Peptides were separated over a 90 min 7%–55% B phase linear gradient (A phase: 2% acetonitrile and 0.1% formic acid; B phase: 80% acetonitrile and 0.1% formic acid) with a flow rate of 300 nl/min by UltiMate 3,000 HPLC system (Thermo Scientific Dionex, USA) and applied on-line to a Velos Pro (Thermo Scientific, USA) dual-pressure ion-trap mass spectrometer. The nano-electrospray ion source (Nanospray Flex, Thermo Scientific, USA) parameters consisted of ion spray voltage 1.7 kV and capillary temperature 250 °C. Spectra were collected over a full scan mode (400–1,500 Da) followed by one higher energy collisional dissociation (HCD) of the five most intense ions from the preceding survey’s full scan under dynamic exclusion criteria ^21^.

### Bioinformatic and statistical analyses

Echocardiographic, hemodynamic, and morphometric data were analyzed using StatSoft STATISTICA 13.5 software for Windows (StatSoft Inc, Tulsa, OK). The data are presented as mean values with the corresponding standard deviations (SD). The Shapiro–Wilk test was used to determine whether quantitative data were normally distributed. Comparisons were performed using t- or Mann–Whitney test for two groups depending on normality. The statistical significance (*p* < 0.05) was calculated with the Bonferroni step-down adjustment to correct the p-value.

The proteomic spectra were analyzed by the X!Tandem (The Global Proteome Machine Organization) and Comet search algorithms and then validated with Peptide Prophet and iProphet under Trans-Proteomic Pipeline software (Institute for Systems Biology, USA). Search parameters consisted of several aspects: (1) taxonomy: rat (UniProtKB/Swiss-Prot); (2) enzyme: trypsin; (3) missed cleavage sites allowed: 2; (4) fixed modification: Methylthio(C); (5) variable modifications: methionine oxidation(M); (6) iTRAQ8plex(K), iTRAQ8plex(N-term), iTRAQ8plex(Y); (7) parent mass error: 1.5 to + 3.0 Da; and (8) peptide fragment mass tolerance: 0.7 Da. Quantitative information was extracted with Libra software under Trans-Proteomic Pipeline. The peptide false discovery rate was estimated by Mayu (Trans-Proteomic Pipeline), and peptide identifications with false discovery rates < 1% were considered correct matches. DanteR software was used for statistical analysis of iTRAQ-labeled peptides^22^. Briefly, data was log2 transformed and normalized using linear regression. Analysis of variance (ANOVA) was performed at a peptide level and the Benjamini & Hochberg false discovery rate (FDR) correction was used to adjust p-values. The mass spectrometry proteomic data were deposited to the ProteomeXchange Consortium via the PRIDE partner repository with the dataset identifier PXD015896^23^.

In order to visualize protein network and gene ontology (GO) annotations, a ClueGO—plug-in software^24^ was used under the Cytoscape 3.3.0 environment^25^. The pathway enrichment analysis was based on GO ontology terms and the Kyoto Encyclopedia of Genes and Genomes (KEGG) pathway with the kappa-statistical score set to 0.4 and fusion criteria (GO Term Fusion) applied to diminish the redundancy of the terms shared by similar associated proteins^26,27^. The minimum number and percentage of associated proteins were set to 3 and 4%, respectively.

### Ethical approval

This study was approved by the 2nd Local Ethical Committee in Cracow, Poland (No 60/2016) and was performed in accordance to the guidelines from Directive 2010/63/EU of the European Parliament on the protection of animals used for scientific purposes.

## Results

### In vivo echocardiographic and hemodynamic measurements

Echocardiographic and hemodynamic parameters measured on sacrifice days are presented in Table 1. Recorded heart rate of animals was significantly higher in early PAH group compared to matched controls (515 ± 26.5 vs. 459 ± 52.9 bpm, *p* = 0.018). The heart rate of end-stage PAH animals was lower compared to matched controls, but this difference was statistically insignificant (434 ± 38.1 vs. 468 ± 57.2 bpm, *p* = 0.184). Echocardiographic measurements show significant thickening of the RV free wall in both early PAH and end-stage PAH animals as compare to controls (0.77 ± 0.04 vs. 0.57 ± 0.08 mm and 1.03 ± 0.09 vs. 0.66 ± 0.03 mm, respectively, *p* < 0.001). Both TAPSE and PAAT/CL values show development of significant pulmonary hypertension in end-stage PAH group (Table 1). Supplementary Table 1 shows echocardiographic measurements recorded during the whole experiment.

**Table 1:** Echocardiographic, hemodynamic and morphometric parameters measured at euthanasia day (mean ± SD).

Parameter	Early PAH rats (n = 8)	Non-PAH matched control rats (n = 8)	*p* value	End-stage PAH rats (n = 8)	Non-PAH matched control rats (n = 8)	*p* value
**Echocardiographic measurements**						
Heart rate (bpm)	515 ± 26.5	459 ± 52.9	**0.018**	434 ± 38.1	468 ± 57.2	0.184
RVFWTd (mm)	0.77 ± 0.04	0.57 ± 0.08	** < 0.001**	1.03 ± 0.09	0.66 ± 0.03	** < 0.001**
TAPSE (mm)	1.02 ± 0.13	1.43 ± 0.57	0.067	0.76 ± 0.13	1.21 ± 0.40	**0.009**
PAAT/CL	0.21 ± 0.06	0.23 ± 0.07	0.549	0.15 ± 0.06	0.22 ± 0.05	**0.024**
**Hemodynamic measurements**						
RV systolic pressure (mmHg)	27.00 ± 4.93	18.43 ± 5.38	**0.005**	50.50 ± 11.56	21.57 ± 2.76	** < 0.001**
RV diastolic pressure (mmHg)	8.43 ± 1.62	5.43 ± 2.64	**0.016**	5.00 ± 2.25	5.25 ± 1.75	0.808
LV systolic pressure (mmHg)	86.14 ± 7.58	90.71 ± 14.61	0.445	45.63 ± 9.10	93.00 ± 15.28	** < 0.001**
LV diastolic pressure (mmHg)	8.71 ± 2.29	10.57 ± 5.22	0.371	6.25 ± 2.60	9.86 ± 5.49	0.115
**Morphometric measurements**						
RV free wall weight (g)	0.21 ± 0.03	0.18 ± 0.02	**0.034**	0.36 ± 0.05	0.16 ± 0.04	** < 0.001**
LV free wall weight (g)	0.35 ± 0.02	0.38 ± 0.05	0.137	0.25 ± 0.02	0.38 ± 0.07	** < 0.001**

*LV* left ventricle, *PAAT/CL* pulmonary artery acceleration time normalized to cycle length, *PAH* pulmonary arterial hypertension, *RV* right ventricle, *RVFWTd* end-diastolic right ventricular free wall thickness, *TAPSE* tricuspid annular plane systolic excursion.

Statistically significant *p* values are given in bold.

Obtained RV hemodynamic parameters indicate development of mild pulmonary hypertension in early PAH group (RV systolic pressure: 27.00 ± 4.93 vs. 18.43 ± 5.38 mmHg; *p* = 0.005) and severe pulmonary hypertension in the end-stage PAH group (50.50 ± 11.56 vs. 21.57 ± 2.76 mmHg; *p* < 0.001). Moreover, impaired systolic function of the LV was noticed in end-stage PAH rats (LV systolic pressure: 45.63 ± 9.10 vs. 93.00 ± 15.28 mmHg; *p* < 0.001). No statistically significant differences in LV diastolic pressures were detected (Table 1).

### Morphometric measurements

Measurements of LV and RV free wall weights on sacrifice days indicated a significant increase in RV myocardium mass in both early and end-stage PAH rats (0.21 ± 0.03 vs. 0.18 ± 0.02 g; *p* = 0.034 and 0.36 ± 0.05 vs. 0.16 ± 0.04 g; *p* < 0.001, respectively). Significant decrease in LV myocardium mass in the end-stage PAH group was also observed (0.25 ± 0.02 versus 0.38 ± 0.07 g; *p* < 0.001).

### Early PAH myocardial protein abundance changes

**Figure 1:**
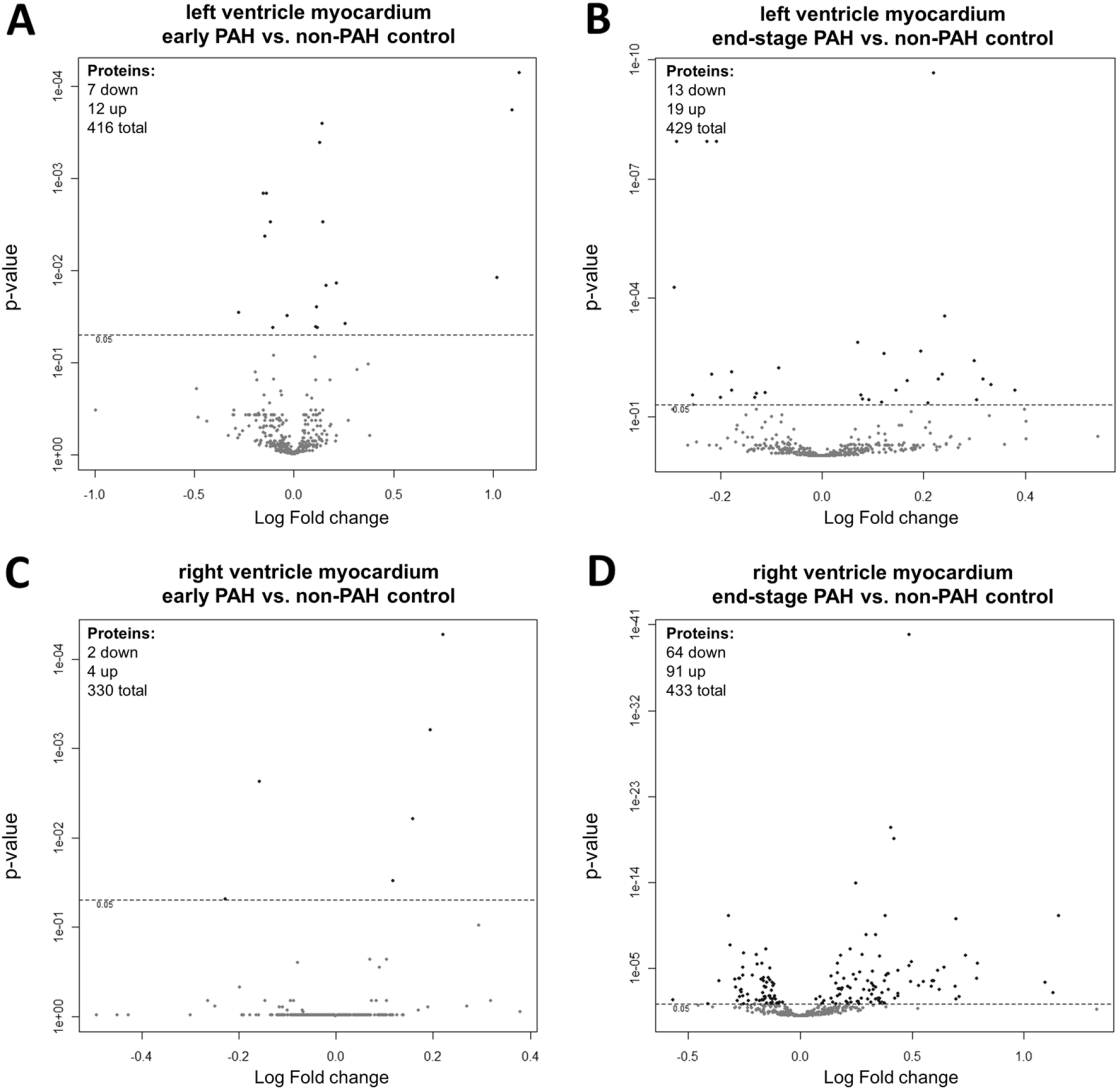
The Volcano Plot of proteins expression in (**A**) left ventricle myocardium of rats with early PAH and (**B**) end-stage PAH, as well as in (**C**) right ventricle myocardium of rats with early PAH and (**D**) end-stage PAH. The graph shows the log2 fold change of protein expression versus p-value. The dashed line indicates threshold 0.05 for *p* value (n = 4 per group).

Changes in protein abundances of LV and RV myocardia collected from rats with end-stage PAH were more meaningful than in subjects with early PAH. The results were presented as Volcano plots based on log2 fold changes and p-values (Fig. 1). Collectively, compared to non-PAH control animals, 19 and six proteins were differentially expressed in RV and LV of rats in the monocrotaline model of PAH at early stage of the disease, respectively (Table 2). In the early PAH group, levels of all fibrinogen chains (alpha, beta, and gamma) were twofold higher in the samples collected from LV myocardia than from the control group. Moreover, serine protease inhibitors (SERPINA3K and A3L), beta-enolase, and mitochondrial enzymes (especially mitochondrial NADP^+^-dependent isocitrate dehydrogenase) were upregulated in the early PAH LV myocardial samples. On the other hand, ezrin was significantly downregulated. Also, the abundance of proteins associated with the glycolytic process (L-lactate dehydrogenase A chain [LDHA] and phosphoglycerate kinase 1 [PGK1]) in addition to myocardial structural proteins (myosin and desmin) decreased in these samples (Table 2). The early proteomic changes in the RV myocardium included an increase in myosin-7 and mitochondrial catabolic pathways (especially fatty acid beta-oxidation) in addition to a decrease in L-lactate dehydrogenase A and protein/nucleic acid deglycase DJ-1 proteins abundance (Table 2). Four of the observed proteins were altered in both RA and LV samples at early PAH stage and have expressed the same direction of change with similar strength (increase in Myosin-7, Methylmalonate-semialdehyde dehydrogenase, Long-chain specific acyl-CoA dehydrogenase and decrease in LDHA) (Table 2).

**Table 2:** Differentially expressed proteins in left and right ventricle myocardium of rats with early PAH (monocrotaline-induced) as compared to control non-PAH animals (*p* < 0.05, n = 4 per group).

Sample	UniProtKB ID	Gene name	Protein name	Fold change	Main biological process associated with the protein
Left ventricle myocardium	P14480	*Fgb*	Fibrinogen beta chain	2.19	Blood coagulation, adaptive immune response, acute-phase response, negative regulation of apoptotic process
	P06399	*Fga*	Fibrinogen alpha chain	2.14	
	P02680	*Fgg*	Fibrinogen gamma chain	2.03	
	P05544	*Serpina3l*	Serine protease inhibitor A3L	1.20	Negative regulation of endopeptidase activity, cell protection from oxidative stress-induced cell death, acute-phase response
	P05545	*Serpina3k*	Serine protease inhibitor A3K	1.16	
	P02564	*Myh7*	Myosin-7	1.12	Fundamental contractile unit of cardiac muscle
	Q9QZ76	*Mb*	Myoglobin	1.11	response to hypoxia, facilitates the movement of oxygen within cardiomyocytes
	P56574	*Idh2*	Isocitrate dehydrogenase [NADP], mitochondrial	1.10	Intermediary metabolism and energy production, glyoxylate cycle
	P15650	*Acadl*	Long-chain specific acyl-CoA dehydrogenase, mitochondrial	1.09	Catalyzes the first step of mitochondrial fatty acid beta-oxidation pathway
	P13803	*Etfa*	Electron transfer flavoprotein subunit alpha, mitochondrial	1.09	Mitochondrial fatty acid beta-oxidation pathway, amino acid metabolism
	Q02253	*Aldh6a1*	Methylmalonate-semialdehyde dehydrogenase [acylating], mitochondrial	1.08	Valine and pyrimidine metabolism, binds fatty acyl-CoA
	P15429	*Eno3*	Beta-enolase	1.08	Glycolytic process, striated muscle development and regeneration
	P02563	*Myh6*	Myosin-6	−1.02	Cardiac muscle contraction
	P48675	*Des*	Desmin	−1.07	Intermediate filament organization
	P08733	*Myl2*	Myosin regulatory light chain 2, ventricular/cardiac muscle isoform	−1.08	Cardiac muscle contraction
	P16617	*Pgk1*	Phosphoglycerate kinase 1	−1.10	Glycolytic pathway
	P16409	*Myl3*	Myosin light chain 3	−1.11	Regulation of cardiac muscle contraction
	P04642	*Ldha*	L-lactate dehydrogenase A chain	−1.11	Lactate metabolic process, positive regulation of apoptotic process
	P31977	*Ezr*	Ezrin	−1.21	Actin cytoskeleton reorganization
Right ventricle myocardium	P02564	*Myh7*	Myosin-7	1.17	Fundamental contractile unit of cardiac muscle
	P17764	*Acat1*	Acetyl-CoA acetyltransferase, mitochondrial	1.14	Catalyzes the last step of mitochondrial fatty acid beta-oxidation pathway
	Q02253	*Aldh6a1*	Methylmalonate-semialdehyde dehydrogenase [acylating], mitochondrial	1.12	Valine and pyrimidine metabolism, binds fatty acyl-CoA
	P15650	*Acadl*	Long-chain specific acyl-CoA dehydrogenase, mitochondrial	1.09	Catalyzes the first step of mitochondrial fatty acid beta-oxidation pathway
	P04642	*Ldha*	L-lactate dehydrogenase A chain	−1.12	Lactate metabolic process, positive regulation of apoptotic process
	O88767	*Park7*	Protein/nucleic acid deglycase DJ-1	−1.17	major nucleotide repair system, regulation of: cell death, apoptotic process, autophagy, oxidative stress

### End-stage-PAH myocardial protein abundance changes

**Figure 2:**
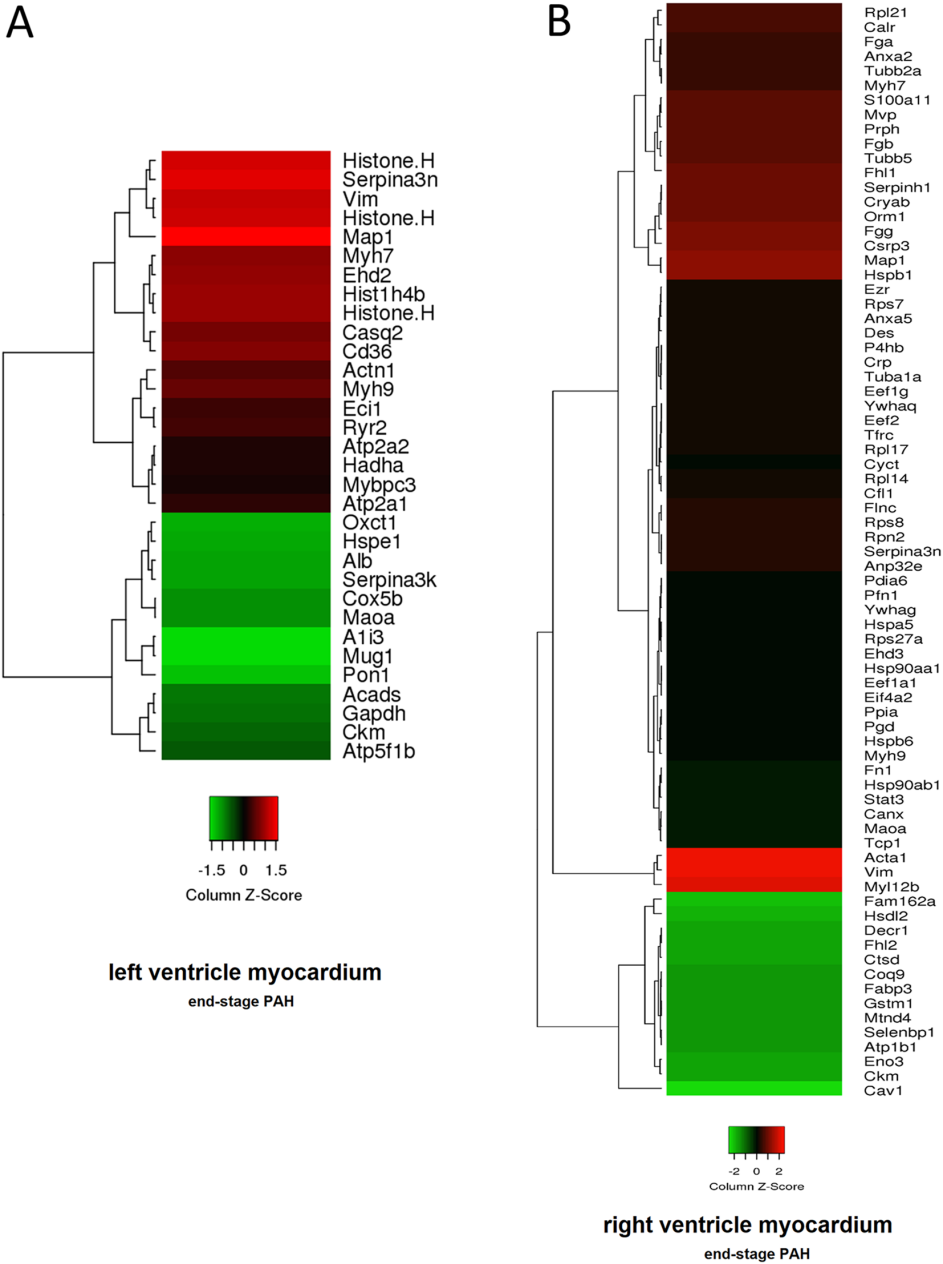
Heat map presentation of a hierarchical cluster of significantly changed proteins in (**A**) left ventricle myocardium (*p* < 0.05; n = 4) and (**B**) right ventricle myocardium (selected with fold change > 1.20 and < −1.20; *p* < 0.05; n = 4) of rats with end-stage monocrotaline-induced PAH. The green and red colors represent low and high expression levels, respectively.

**Figure 3:**
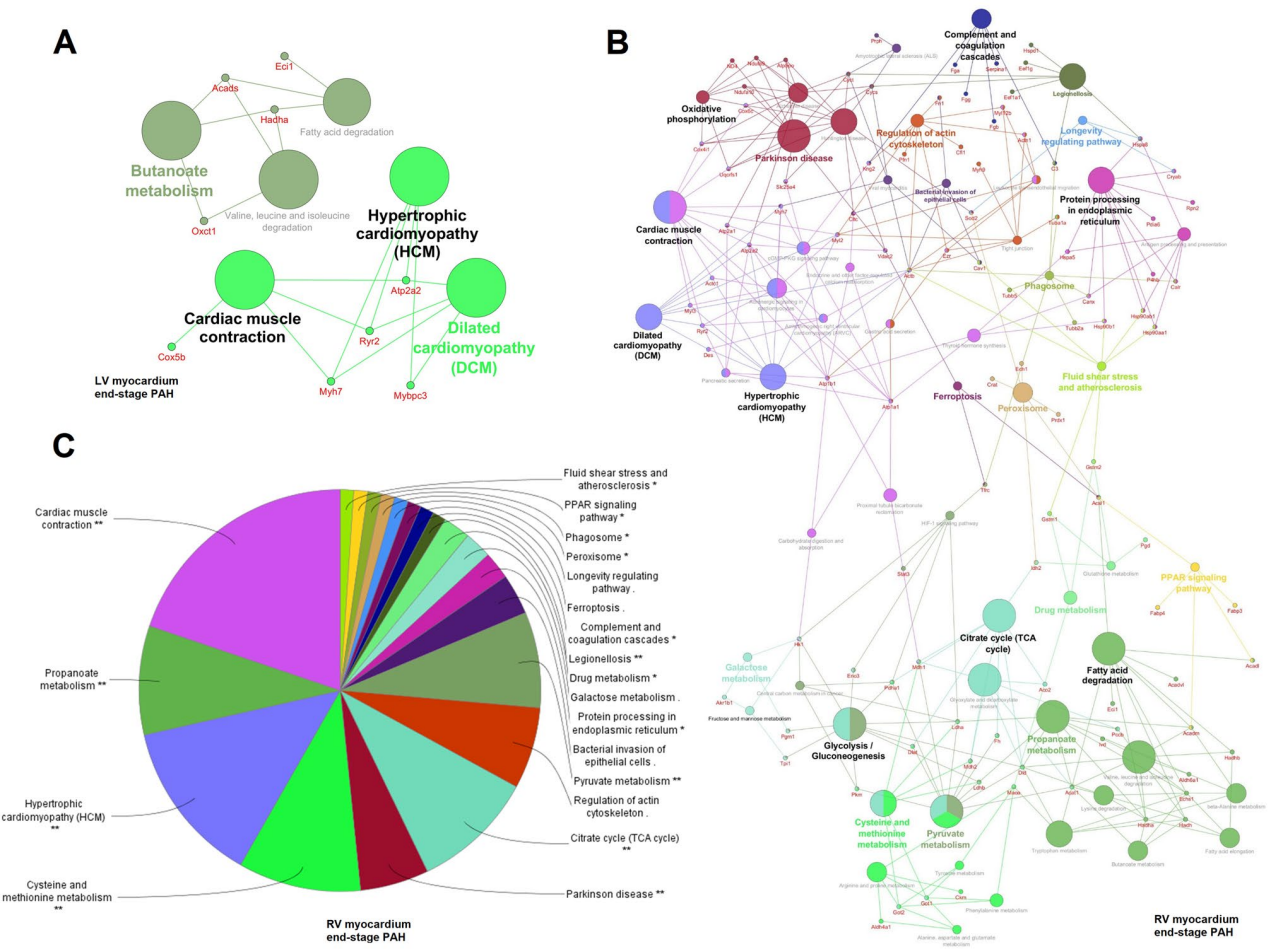
Enriched GO network related to KEGG pathways (https://www.kegg.jp/kegg/pathway.html) in (**A**) left ventricle myocardium of rats and (**B**) right ventricle myocardium of rats in end-stage monocrotaline-induced PAH. (**C**) KEGG pathways significantly enriched in right ventricle myocardium of rats with end-stage PAH depicted as a circle chart (*p* < 0.05). Biological processes and genes shared between pathways in left/right ventricle were visualized with ClueGO (kappa score ≥ 0.4) under the Cytoscape 3.3.0 environment as a functional grouped network. Each node represents a GO term or a gene. The enrichment significance of the GO terms is reflected by the size of the nodes. Edges represent connections between the nodes. (ClueGO under the Cytoscape 3.3.0 environment, https://apps.cytoscape.org/apps/cluego).

At the end-stage of PAH, 32 and 155 proteins were significantly changed in LV and RV, respectively (Tables 3 and 4). Changes in protein abundances in rats with end-stage PAH were displayed as heat maps, that show a hierarchical cluster of differentially expressed proteins in RV and LV (Fig. 2). In order to examine the biological processes that play important roles in RV and LV remodeling in PAH, we performed pathway enrichment analyses using a ClueGO software under the Cytoscape 3.3.0 environment. In the LV of rats with end-stage PAH, we have observed enriched pathways related to cardiac muscle contraction and cardiomyopathies (Fig. 3A). Especially, an increased t-kininogen 1, vimentin, and Ca^2+^ ion-related proteins (ryanodine receptor 2, calsequestrin-2, and sarco/endoplasmic reticulum Ca^2+-^ATPase [SERC-1 and -2]) abundance should be noticed (Table 3).

**Table 3:** Differentially expressed proteins in left ventricle myocardium of rats with end-stage PAH (monocrotaline-induced) as compared to control non-PAH animals (*p* < 0.05, n = 4 per group).

UniProtKB ID	Gene name	Protein name	Fold change
P01048	*Map1*	T-kininogen 1	1.30
P09006	*Serpina3n*	Serine protease inhibitor A3N	1.26
Q6LED0	*n/a*	Histone H3.1	1.25
Q64598	*n/a*	Histone H2A type 1-F	1.24
P31000	*Vim*	Vimentin	1.23
Q00715	*n/a*	Histone H2B type 1	1.18
P62804	*Hist1h4b*	Histone H4	1.18
Q4V8H8	*Ehd2*	EH domain-containing protein 2	1.17
P02564	*Myh7*	Myosin-7	1.16
Q07969	*Cd36*	Platelet glycoprotein 4	1.16
P51868	*Casq2*	Calsequestrin-2	1.14
Q62812	*Myh9*	Myosin-9	1.12
Q9Z1P2	*Actn1*	Alpha-actinin-1	1.11
B0LPN4	*Ryr2*	Ryanodine receptor 2	1.09
P23965	*Eci1*	Enoyl-CoA delta isomerase 1, mitochondrial	1.09
Q64578	*Atp2a1*	Sarcoplasmic/endoplasmic reticulum calcium ATPase 1 (SERCA1)	1.07
Q64428	*Hadha*	Trifunctional enzyme subunit alpha, mitochondrial	1.06
P11507	*Atp2a2*	Sarcoplasmic/endoplasmic reticulum calcium ATPase 2 (SERCA2)	1.06
P56741	*Mybpc3*	Myosin-binding protein C, cardiac-type	1.05
P10719	*Atp5f1b*	ATP synthase subunit beta, mitochondrial	−1.06
P00564	*Ckm*	Creatine kinase M-type	−1.08
P04797	*Gapdh*	Glyceraldehyde-3-phosphate dehydrogenase	−1.09
P15651	*Acads*	Short-chain specific acyl-CoA dehydrogenase, mitochondrial	−1.10
P21396	*Maoa*	Amine oxidase [flavin-containing] A	−1.13
P12075	*Cox5b*	Cytochrome c oxidase subunit 5B, mitochondrial	−1.13
P05545	*Serpina3k*	Serine protease inhibitor A3K	−1.15
P02770	*Alb*	Serum albumin	−1.16
P26772	*Hspe1*	10 kDa heat shock protein, mitochondrial	−1.16
B2GV06	*Oxct1*	Succinyl-CoA:3-ketoacid coenzyme A transferase 1, mitochondrial	−1.17
P55159	*Pon1*	Serum paraoxonase/arylesterase 1	−1.19
Q03626	*Mug1*	Murinoglobulin-1	−1.22
P14046	*A1i3*	Alpha-1-inhibitor 3	−1.22

**Table 4:** Differentially expressed proteins in right ventricle myocardium of rats with end-stage PAH (monocrotaline-induced) as compared to control non-PAH animals (*p* < 0.05, n = 4 per group).

UniProtKB ID	Gene name	Protein name	Fold change	UniProtKB ID	Gene name	Protein name	Fold change
P31000	*Vim*	Vimentin	2.23	P62630	*Eef1a1*	Elongation factor 1-alpha 1	1.26
P68136	*Acta1*	Actin, alpha skeletal muscle	2.19	P82995	*Hsp90aa1*	Heat shock protein HSP 90-alpha	1.26
P18666	*Myl12b*	Myosin regulatory light chain 12B	2.14	Q8R491	*Ehd3*	EH domain-containing protein 3	1.26
P42930	*Hspb1*	Heat shock protein beta-1	1.73	P62982	*Rps27a*	Ubiquitin-40S ribosomal protein S27a	1.25
P01048	*Map1*	T-kininogen 1	1.73	P06761	*Hspa5*	Endoplasmic reticulum chaperone BiP	1.25
P50463	*Csrp3*	Cysteine and glycine-rich protein 3	1.67	P62963	*Pfn1*	Profilin-1	1.25
P02680	*Fgg*	Fibrinogen gamma chain	1.64	Q63081	*Pdia6*	Protein disulfide-isomerase A6	1.25
P02764	*Orm1*	Alpha-1-acid glycoprotein	1.62	P61983	*Ywhag*	14-3-3 protein gamma	1.25
P23928	*Cryab*	Alpha-crystallin B chain	1.62	P97541	*Hspb6*	Heat shock protein beta-6	1.24
P29457	*Serpinh1*	Serpin H1	1.62	P85968	*Pgd*	6-phosphogluconate dehydrog., decarboxylating	1.23
Q9WUH4	*Fhl1*	Four and a half LIM domains protein 1	1.56	P10111	*Ppia*	Peptidyl-prolyl cis–trans isomerase A	1.23
P69897	*Tubb5*	Tubulin beta-5 chain	1.54	Q62812	*Myh9*	Myosin-9	1.23
P14480	*Fgb*	Fibrinogen beta chain	1.53	P52631	*Stat3*	Signal transducer and activator of transcription 3	1.22
P21807	*Prph*	Peripherin	1.51	P34058	*Hsp90ab1*	Heat shock protein HSP 90-beta	1.21
Q62667	*Mvp*	Major vault protein	1.51	P04937	*Fn1*	Fibronectin	1.21
Q6B345	*S100a11*	Protein S100-A11	1.50	P21396	*Maoa*	Amine oxidase [flavin-containing] A	1.20
P18418	*Calr*	Calreticulin	1.47	P35565	*Canx*	Calnexin	1.20
P20280	*Rpl21*	60S ribosomal protein L21	1.44	P28480	*Tcp1*	T-complex protein 1 subunit alpha	1.20
Q07936	*Anxa2*	Annexin A2	1.41	P62250	*Rps16*	40S ribosomal protein S16	1.19
P06399	*Fga*	Fibrinogen alpha chain	1.41	P20059	*Hpx*	Hemopexin	1.19
P02564	*Myh7*	Myosin-7	1.40	P60711	*Actb*	Actin, cytoplasmic 1	1.19
P85108	*Tubb2a*	Tubulin beta-2A chain	1.40	P05708	*Hk1*	Hexokinase-1	1.18
Q5XIE0	*Anp32e*	Acidic leucine-rich nuclear phosphoprotein 32 family member E	1.35	Q5XFX0	*Tagln2*	Transgelin-2	1.17
P09006	*Serpina3n*	Serine protease inhibitor A3N	1.35	Q63041	*A1m*	Alpha-1-macroglobulin	1.17
P25235	*Rpn2*	Dolichyl-diphosphooligosaccharide–protein glycosyltransferase subunit 2	1.35	Q6LED0	*n/a*	Histone H3.1	1.17
P62243	*Rps8*	40S ribosomal protein S8	1.34	Q66HD0	*Hsp90b1*	Endoplasmin	1.17
D3ZHA0	*Flnc*	Filamin-C	1.34	P62804	*Hist1h4b*	Histone H4	1.16
P48675	*Des*	Desmin	1.32	P0DMW1	*Hspa1b*	Heat shock 70 kDa protein 1B	1.16
P62083	*Rps7*	40S ribosomal protein S7	1.32	P70567	*Tmod1*	Tropomodulin-1	1.15
P31977	*Ezr*	Ezrin	1.31	Q9Z1P2	*Actn1*	Alpha-actinin-1	1.14
P14668	*Anxa5*	Annexin A5	1.31	P17475	*Serpina1*	Alpha-1-antiproteinase	1.14
Q68FR6	*Eef1g*	Elongation factor 1-gamma	1.30	A0JPQ4	*Trim72*	Tripartite motif-containing protein 72	1.14
P68370	*Tuba1a*	Tubulin alpha-1A chain	1.30	P08733	*Myl2*	Myosin regulatory light chain 2, ventricular/cardiac isoform	1.14
P48199	*Crp*	C-reactive protein	1.30	P01026	*C3*	Complement C3	1.13
P04785	*P4hb*	Protein disulfide-isomerase	1.30	P62898	*Cycs*	Cytochrome c, somatic	1.13
P45592	*Cfl1*	Cofilin-1	1.29	P11442	*Cltc*	Clathrin heavy chain 1	1.13
Q63507	*Rpl14*	60S ribosomal protein L14	1.29	Q00715	*Hist1h2bl*	Histone H2B type 1	1.12
P24049	*Rpl17*	60S ribosomal protein L17	1.28	P04642	*Ldha*	L-lactate dehydrogenase A chain	1.12
P05197	*Eef2*	Elongation factor 2	1.28	P63018	*Hspa8*	Heat shock cognate 71 kDa protein	1.12
P68255	*Ywhaq*	14–3-3 protein theta	1.28	P63102	*Ywhaz*	14–3-3 protein zeta/delta	1.12
Q99376	*Tfrc*	Transferrin receptor protein 1	1.28	P16409	*Myl3*	Myosin light chain 3	1.12
P10715	*Cyct*	Cytochrome c, testis-specific	1.27	P38652	*Pgm1*	Phosphoglucomutase-1	1.11
Q5RKI1	*Eif4a2*	Eukaryotic initiation factor 4A-II	1.26	P68035	*Actc1*	Actin, alpha cardiac muscle 1	1.10
Q63716	*Prdx1*	Peroxiredoxin-1	1.10	P10888	*Cox4i1*	Cytochrome c oxidase subunit 4 isoform 1, mitochondrial	−1.12
Q5BK63	*Ndufa9*	NADH dehydrogenase [ubiquinone] 1 α subunit 9, mitochondrial	1.08	P81155	*Vdac2*	Voltage-dependent anion-selective channel protein 2	−1.12
P30427	*Plec*	Plectin	1.07	Q60587	*Hadhb*	Trifunctional enzyme subunit beta, mitochondrial	-1.12
P63039	*Hspd1*	60 kDa heat shock protein, mit	1.06	Q02253	*Aldh6a1*	Methylmalonate-semialdehyde dehydrogenase [acylating], mit	−1.12
P11980	*Pkm*	Pyruvate kinase PKM	1.05	P23965	*Eci1*	Enoyl-CoA delta isomerase 1, mit	−1.12
B0LPN4	*Ryr2*	Ryanodine receptor 2	−1.06	P08461	*Dlat*	Dihydrolipoyllysine-residue acetyltransferase component of pyruvate dehydrog. complex, mit	−1.12
P48500	*Tpi1*	Triosephosphate isomerase	−1.07	P11530	*Dmd*	Dystrophin	−1.12
P08503	*Acadm*	Medium-chain specific acyl-CoA dehydrogenase, mitochondrial	−1.07	O88989	*Mdh1*	Malate dehydrogenase, cytoplasmic	−1.13
P06685	*Atp1a1*	Sodium/potassium-transporting ATPase subunit alpha-1	−1.08	P20788	*Uqcrfs1*	Cytochrome b-c1 complex subunit Rieske, mitochondrial	−1.13
P16036	*Slc25a3*	Phosphate carrier protein, mitochondrial	−1.08	P17764	*Acat1*	Acetyl-CoA acetyltransferase, mitochondrial	−1.14
P13221	*Got1*	Aspartate aminotransferase, cytoplasmic	−1.08	P56574	*Idh2*	Isocitrate dehydrogenase [NADP], mitochondrial	−1.14
P04636	*Mdh2*	Malate dehydrogenase, mitochondrial	−1.09	P08010	*Gstm2*	Glutathione S-transferase Mu 2	−1.15
P45953	*Acadvl*	Very long-chain specific acyl-CoA dehydrogenase, mitochondrial	−1.09	Q64578	*Atp2a1*	Sarcoplasmic/endoplasmic reticulum calcium ATPase 1	−1.16
Q05962	*Slc25a4*	ADP/ATP translocase 1	−1.09	Q704S8	*Crat*	Carnitine O-acetyltransferase	−1.17
P07633	*Pccb*	Propionyl-CoA carboxylase beta chain, mitochondrial	−1.09	P70623	*Fabp4*	Fatty acid-binding protein, adipocyte	−1.18
P42123	*Ldhb*	L-lactate dehydrogenase B chain	−1.09	P0C2X9	*Aldh4a1*	Delta-1-pyrroline-5-carboxylate dehydrogenase, mitochondrial	−1.19
Q561S0	*Ndufa10*	NADH dehydrogenase [ubiquinone] 1 alpha subcomplex subunit 10, mit	−1.09	Q9QZ76	*Mb*	Myoglobin	−1.19
Q3KR86	*Immt*	MICOS complex subunit Mic60	−1.09	Q9WVK7	*Hadh*	Hydroxyacyl-coenzyme A dehydrogenase, mitochondrial	−1.19
P14408	*Fh*	Fumarate hydratase, mitochondrial	−1.09	P11951	*Cox6c2*	Cytochrome c oxidase subunit 6C-2	−1.19
P14604	*Echs1*	Enoyl-CoA hydratase, mitochondrial	−1.10	P07895	*Sod2*	Superoxide dismutase [Mn], mitochondrial	−1.19
Q9ER34	*Aco2*	Aconitate hydratase, mitochondrial	−1.10	P07340	*Atp1b1*	Sodium/potassium-transporting ATPase subunit beta-1	−1.20
P12007	*Ivd*	Isovaleryl-CoA dehydrogenase, mit	−1.10	Q8VIF7	*Selenbp1*	Methanethiol oxidase	−1.20
P18163	*Acsl1*	Long-chain-fatty-acid-CoA ligase 1	−1.10	P05508	*Mtnd4*	NADH-ubiquinone oxidoreductase chain 4	−1.20
P26284	*Pdha1*	Pyruvate dehydrogenase E1 component subunit alpha, mit	−1.10	P04905	*Gstm1*	Glutathione S-transferase Mu1	−1.20
Q06647	*Atp5po*	ATP synthase subunit O, mitochondrial	−1.10	P07483	*Fabp3*	Fatty acid-binding protein, heart	−1.21
Q6P6R2	*Dld*	Dihydrolipoyl dehydrogenase, mit	−1.10	Q68FT1	*Coq9*	Ubiquinone biosynthesis protein COQ9, mitochondrial	−1.21
Q9Z0V6	*Prdx3*	Thioredoxin-dependent peroxide reductase, mitochondrial	−1.11	P24268	*Ctsd*	Cathepsin D	−1.22
P15650	*Acadl*	Long-chain specific acyl-CoA dehydrogenase, mitochondrial	−1.11	O35115	*Fhl2*	Four and a half LIM domains protein 2	−1.22
Q64428	*Hadha*	Trifunctional enzyme subunit alpha, mitochondrial	−1.11	Q64591	*Decr1*	2,4-dienoyl-CoA reductase, mitochondrial	−1.22
P00507	*Got2*	Aspartate aminotransferase, mit	−1.11	P00564	*Ckm*	Creatine kinase M-type	−1.24
Q6UPE1	*Etfdh*	Electron transfer flavoprotein-ubiquinone oxidoreductase, mitochondrial	−1.11	P15429	*Eno3*	Beta-enolase	−1.25
Q62651	*Ech1*	Delta(3,5)-Delta(2,4)-dienoyl-CoA isomerase, mitochondrial	−1.11	Q4V8F9	*Hsdl2*	Hydroxysteroid dehydrogenase-like protein 2	−1.29
P07943	*Akr1b1*	Aldose reductase	−1.11	Q4QQV3	*Fam162a*	Protein FAM162A	−1.33
P11507	*Atp2a2*	Sarcoplasmic/endoplasmic reticulum calcium ATPase 2	−1.12	P41350	*Cav1*	Caveolin-1	−1.48
P39069	*Ak1*	Adenylate kinase isoenzyme 1	−1.12

In the RV of rats with end-stage PAH, we found enriched pathways connected to cardiac muscle contraction, hypertrophic cardiomyopathy, and dilated cardiomyopathy as well as other processes related to Krebs cycle, glycolysis, pyruvate metabolism, fatty acid degradation, oxidative phosphorylation, protein processing in the endoplasmic reticulum, and complement and coagulation cascades (Fig. 3B, C). Importantly, in PAH-induced RV remodeling, we observed upregulated structural proteins (such as: actin, myosin, desmin, tubulin, filamin), regulatory proteins (especially major vault protein, annexin A2, ezrin, 14-3-3 protein, profilin 1, peptidyl-prolyl cis–trans isomerase A, STAT3, transgelin-2, complement C3, HSP 90) and proteins responsible for protein processing in the endoplasmic reticulum (such as calreticulin, calnexin, heat shock proteins, endoplasmic reticulum chaperone BiP) in addition to protein synthesis (such as 40/60S ribosomal proteins, elongation factors) or fibrosis (fibronectin and vimentin). These changes were accompanied by the significant downregulation of caveolin-1 and FAM162A. Finally, proteins associated with fatty acid beta-oxidation pathway (enoyl-CoA hydratase, long-chain specific acyl-CoA dehydrogenase, hydroxyacyl-coenzyme A dehydrogenase) were decreased compared to non-PAH controls.

Sixteen of the observed proteins were altered in both RA and LV samples at end-stage PAH, among which 10 have expressed the same direction of change. However, substantial difference was found in Ca^2+^ ion-related proteins abundance (ryanodine receptor 2, SERC-1 and SERC-2), which were upregulated in LV and downregulated in RV samples of rats with end-stage PAH (Tables 3 and 4). Supplementary Table 1 shows abundance of LV and RV myocardium proteins that are significantly altered in both early and end-stage PAH.

### Histological analysis

**Figure 4:**
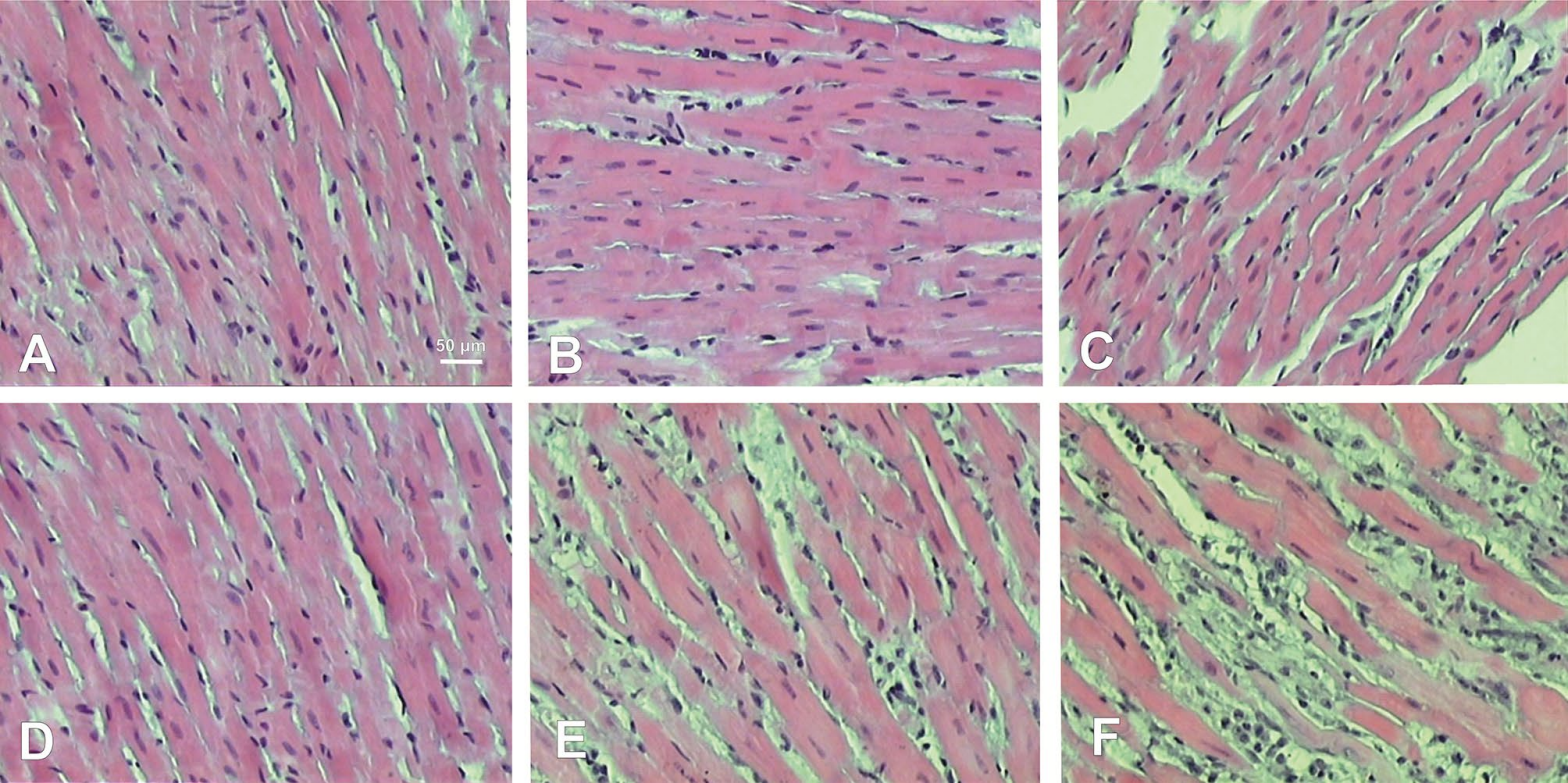
Histological cross-sections (hematoxylin and eosin staining) of left (**A**–**C**) and right (**D**–**F**) ventricle samples showing different stages of PAH development. A—left ventricle non-PAH control group, B—left ventricle early PAH, C—left ventricle end-stage PAH, D—right ventricle non-PAH control group, E—right ventricle early PAH, F—right ventricle end-stage PAH.

**Figure 5:**
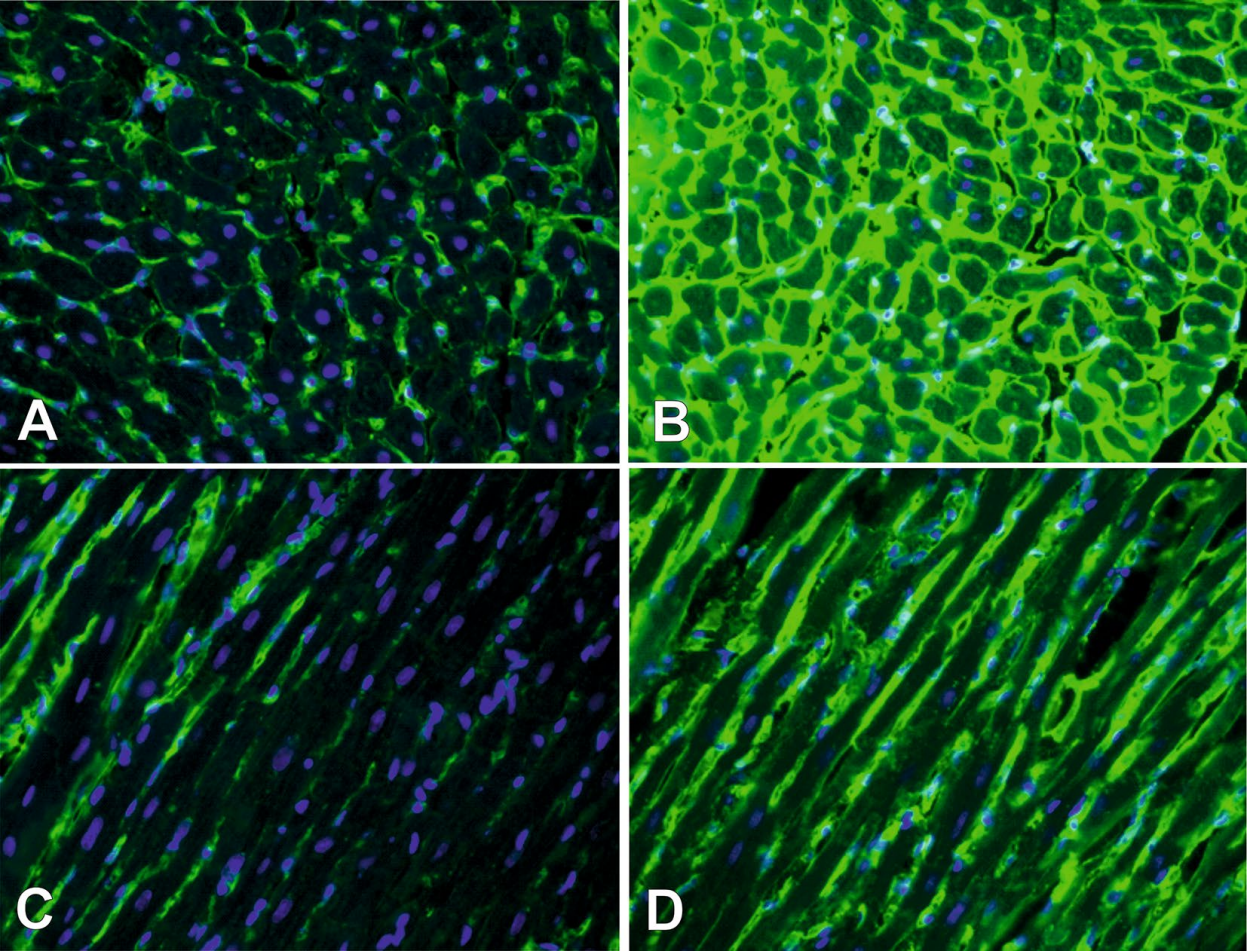
Histological cross-sections (Wheat Germ Agglutinin–Alexa Fluor 488 and DAPI [4,6-diamidino-2-phenylindole hydrochloride] staining) of left ventricle myocardium in non-PAH control group (**A**, **C**) and end-stage PAH animals (**B**, **D**).

Hematoxylin and eosin staining of samples showed significant changes in both LV and RV (Fig. 4). In LV myocardium no considerable structural changes were observed until end-stage PAH, then reduced size of cardiomyocytes and increased connective tissue volume were present in end-stage PAH animals (Fig. 4A–C). In RV samples, visible changes were detected in early PAH that include increased size of cardiomyocytes and increased connective tissue and extracellular matrix volumes as well as inflammatory cells infiltration, that intensified in end-stage PAH group (Fig. 4D–F). Wheat Germ Agglutinin immunofluorescence staining was performed to detect cardiac fibrosis in studied samples, showing significantly increased amount of myocardial fibrotic tissue in both RV and LV samples in end-stage PAH animals, compared to matched controls (Fig. 5).

## Discussion

**Figure 6:**
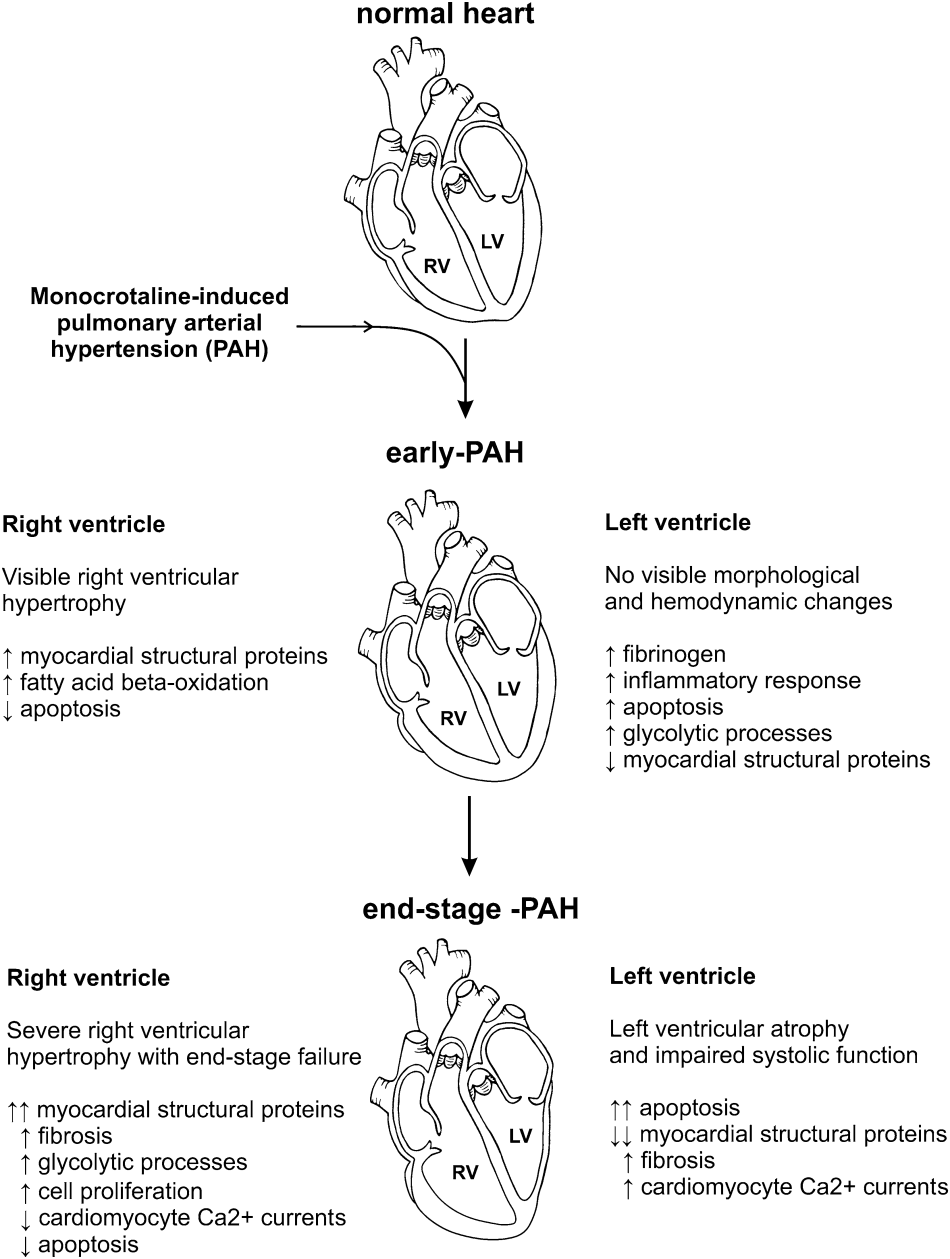
Schematic summary of the results of the study.

In the present study we analyzed the mechanisms of left and right ventricles adaptation and failure in a monocrotaline-induced model of PAH using a proteome-analysis based approach. Especially, we have identified changes in the levels of several proteins, and thus revealing potential metabolic pathways related to response of the heart muscle at the very early stages of PAH that are accompanied by barely expressed RV and no LV macroscopic abnormalities (Fig. 6). This approach and results of our study may contribute to delineation of potential therapeutic targets for the treatment of the PAH.

### LV changes over the PAH

During the early stages of monocrotaline-induced PAH, no significant changes in either LV size or function were observed, whereas at the later stages of PAH, significant LV atrophy was observed. Thus far, two different mechanisms have been proposed to explain PAH related LV atrophy. One of them is a decrease in initial LV load, caused by the increase in pulmonary vasculature resistance which is a trigger for decreased RV stroke volume and thus decreased LV end-diastolic filling (hemodynamic stress). Another possible mechanism includes hypoxia and myocardial ischemia, resulting from RV heart failure (metabolic stress)^28^. Most likely, the PAH-related LV remodeling is the result of many complex mechanisms, starting from the first days of PAH development.

Despite the lack of tangible macroscopic changes, some significant disturbances in LV myocardial protein abundance may be observed during the early PAH stages. The most pronounced changes include an increase in fibrinogen levels, which is also a positive acute-phase protein in addition to being a major coagulation cascade protein^29^. The relationship between increased fibrinogen plasma levels and progression of various types of pulmonary hypertension has been well documented, whereas little is known about its role in the myocardium^30^. We have observed an increase in fibrinogen levels in the LV myocardium, which may indicate the occurrence of two phenomena. First, the accumulation of fibrinogen may be considered an inflammatory response, which is caused by short-lived monocrotaline metabolites directly damaging pulmonary endothelium and myocardium. Second, local activation of coagulation factors and thus thrombosis induction in the myocardial microvasculature may be expected^31^.

Moreover, our study shows significant changes in abundance of proteins engaged in cell death pathway regulation, which may indicate an unstable balance in this matter in the LV myocardium during early PAH stages. For example, we observed an increase in the level of proteins protecting against premature or unwanted activation of apoptosis. These proteins include serpin family A member (SERPINA3, which protects cells from oxidative stress-induced cell death and also serves as an acute phase reactant by inhibiting cathepsin G, which may limit inflammation and coagulation)^32^ and mitochondrial NADP^+^-dependent isocitrate dehydrogenase (its suppression induces apoptosis and hypertrophy of cultured cardiomyocytes) ^33^. On the other hand, ezrin, a negative regulator of death receptor-induced apoptosis^34^ was significantly downregulated in the LV myocardium from early PAH rats. Moreover, in the current study, a significant decrease in levels of proteins associated with glycolytic processes (L-lactate dehydrogenase A chain [LDHA] and phosphoglycerate kinase [PGK]1) that may promote apoptosis were detected as it was proven that LDHA silencing induces an apoptosis via the mitochondrial pathway^35^, and PGK1 repression leads to a decrease in ATP levels, thus accelerating apoptosis^36^. This slowly developing programmed cardiomyocyte death process is reflected in decreasing level of myocardial structural proteins (myosin and desmin).

The above discussed mechanisms initiate structural and functional changes in the LV that may be clearly observed during end-stage PAH, in which functional cardiomyocytes are subject to atrophy and are replaced by fibrous tissue (reflected by increased level of vimentin)^37^. Nevertheless, other significant pathways that may be responsible for LV remodeling may be induced during later stages of the disease. The significant changes were observed in Ca^2+^ ion-related pathways, especially: ryanodine receptor 2 (protein functions as the major component of a calcium channel located in the sarcoplasmic reticulum that supplies ions to the cardiac muscle during systole), calsequestrin-2 (high-capacity, moderate affinity, Ca^2+^-binding protein acting as an internal Ca^2+^ ion store) and SERCA-1 and -2 (Ca^2+^ ATPase that transfers Ca^2+^ ions from the cytosol to the lumen of the sarcoplasmic reticulum at the expense of ATP hydrolysis during muscle relaxation). SERCA proteins cooperate to increase Ca^2+^ movements in cardiomyocytes aiming to increase myocardium contraction. Increased levels of these proteins may suggest that a failing LV with apoptosis-induced reduction in the number of functional cardiomyocytes (and therefore reduced force production) and reduced myosin content (also causing reduction in force production by a reduction in the number of available cross bridges per sarcomere) tries to maintain its function by increasing Ca^2+^ currents. This hypothesis may be confirmed by findings of Pham et al. study which have proved that LV trabeculae from PAH rats maintained normal mechano-energetic performance despite its atrophy^38^.

### RV changes over the PAH

Structural and functional changes in the RV occur at early stages of PAH, long before those observed in LV. The results of current study largely confirm and support the existing molecular mechanisms explaining PAH-induced RV remodeling. Our study found that early pressure overload of the right heart chamber induces an increased synthesis of thick filament proteins, such as myosin-7, which is a protein strongly linked to the hypertrophic cardiomyopathy development^39^, and concurrently inhibits apoptotic and autophagy pathways (decrease in protein/nucleic acid deglycase DJ-1, which is an anti-oxidative and autophagy modulator protein) that further promote cardiac hypertrophy^40^. Moreover, early alterations also include mitochondrial catabolic pathways intensification (especially fatty acid beta-oxidation), which is the answer to the increased energy demand for stressed myocardium^41^.

At the later stages of PAH, RV remodeling progresses and is associated with further increases in cardiomyocyte structural protein synthesis (e.g. actin, myosin, desmin, tubulin, filamin) but also with fibrosis (fibronectin and vimentin). Especially, the latter process contributes to the acceleration of concomitant heart failure after pressure overload; the maladaptive effects of fibronectin make this protein a good target for future therapeutic strategies^42^. Moreover, further metabolic changes are observed, which include switching from oxidative phosphorylation to aerobic glycolysis. Also, downregulation of proteins related to cardiomyocyte Ca^2+^ currents were observed.

Furthermore, we have identified upregulated levels of several important regulatory proteins responsible for RV hypertrophy enhancement that may be considered a potential therapy target. Especially, targeting STAT3, which is indicated as a key mediator of PAH, has the potential to not only inhibit cell proliferation, survival, and motility but also immune escape and altered immunologic environment^43^. The major vault protein (a cell survival factor) together with HSP 90, that is essential for creation, maintenance, and destruction of proteins, also deserve special attention as they may play key roles in cardiovascular pathophysiology. Moreover, both HSP 90 and major vault protein are inhibited by carfilzomib, an anti-tumor drug that was recently found to reverse PAH, which may explain protective effect of the drug^44^. Other promising proteins include profilin 1, which overexpression is sufficient to induce cardiomyocyte hypertrophy and sarcomere remodeling, and silencing attenuates the hypertrophic response^45^. Furthermore, 14-3-3 protein, having an anti-apoptotic role through phosphorylation-dependent binding^46^ and transgelin-2, that is an actin‐binding protein implicated in actin dynamics which induce cell proliferation and migration^47^ are worthy of our attention. Also, we have observed increased abundance of calreticulin, that is an effective inducer of cardiac growth, which activation might be involved in hypoxic signaling leading to pulmonary hypertension; calreticulin activity may be inhibited by cyclosporin A, thus preventing RV hypertrophy^48^. Finally, caveolin-1 protein was observed to be strongly downregulated, which may drive p42/44 MAP kinase activation and cardiac hypertrophy^49^.

Our results are in line with previous observations, although several discrepancies may be observed. Study by Aziz et al. claimed to show both an adaptive and maladaptive RV response to dehydromonocrotaline-induced early chronic pulmonary hypertension in canine model. A significant downregulation of RV proteins involved in contractile function, energy metabolism and protein quality control as well as activation of cellular stress mechanisms were observed^50^. Although authors have demanded that these changes are related to early RV response, they are more consistent with the alterations we have observed at the end stages of the disease. Interestingly, study by Bond et al. showed abnormalities in the calcium signaling pathways of the RV myocardium in children with hypertensive RV, where increased expression of myocardial contractile and extracellular proteins was accompanied by enriched calcium signaling^51^. Same increase in RV structural and contractile proteins were observed in current study however downregulation of proteins related to cardiomyocyte Ca^2+^ currents were noted. Using RV hypertrophy piglet model Sheikh et al. showed significant increase in structural proteins, but a fall in HSP-70 expression, protein that may directly inhibits apoptosis^52^. Meanwhile, the proteins indicated by our study point to suppressed RV apoptosis at all stages of PAH. All these differences may arise from the use of other study models and collection of samples from different disease stages.

### Strengths and limitations

The main strength of our study is an implementation of the global proteome assessment method (iTRAQ), which has several advantages over the other methods (such as RNA sequencing) used for the identification of molecular mechanisms underlying heart-specific changes over the PAH course. In particular, high throughput proteomics is capable of showing the effective presence and amount of functional proteins in studied samples, whereas genomic profiling provides information on the pre-translational level of genetic material that does not fully imply its true correspondence with protein levels or effective activities^53^. Another strength of the study is that, due to its design, we were able to describe a sequence of metabolic and structural changes of the heart ventricles over the course of PAH progression. Moreover, we were able to delineate the profiles of the very early adaptive response of the RV and LV to an increased pulmonary artery pressure at the time of no macroscopic abnormalities.

The main limitation of our study is that the results of animal experiments may not be fully translated into human PAH pathomechanisms. It is well established that monocrotaline has toxic effect, that can be also observed directly on the myocardium and thus proteomic analysis could be biased by this fact. Nonetheless, our study implemented pre-selection protocol that excluded samples with moderate and severe signs of myocarditis, which should endure most of monocrotaline related negative effects in this aspect. Additionally, we may not ignore that some of our observations are specific to the monocrotaline-induced model of PAH and are not relevant for natural course of the PAH in diseased patients. Moreover, not all observed morphological and molecular ventricular changes may result from PAH development, but they could also be a consequence of pulmonary vascular inflammation or neurohumoral activation, that indirectly affect the myocardium. However, it should be emphasized that the monocrotaline rat model is a generally accepted and widely used experimental model of PAH. A heart tissue collected from living PAH humans to assess the early adaptive response of the LV and RV is unobtainable without a significant risk for the patient^12^. Although a female predominance is observed in PAH natural course in humans, only male rats were used in current model. Such a selection of individuals may affect results of our study, mainly due to the different female genotype and presence of female sex hormones^54^. Nevertheless, this is consistent with other studies using only male animals and thus direct between-studies comparisons are possible. Finally, further validation of results presented in this study should be performed to support our findings.

## Conclusion

Significant remodeling of both heart ventricles is observed over the course of monocrotaline-induced PAH. The present study provides new insights into the mechanisms underlying myocardial remodeling at the early and late stage of this disease. LV damage is linked to an increase in apoptotic pathway activity, intensified fibrosis, reduced structural protein levels, switch to glycolytic versus aerobic processes, and alterations in Ca^2+^ homeostasis. RV pressure overload leads to its maladaptive hypertrophy and diverse dilated cardiomyopathy-mediated regulatory pathways.

## Supplementary information

Supplementary file1

## Data Availability

The datasets generated during the current study are available in the ProteomeXchange Consortium via the PRIDE partner repository with the dataset identifier PXD015896 [https://www.ebi.ac.uk/pride/archive].
