# Supplementary material for: Myocardial proteomic profile in pulmonary arterial hypertension
Source: Sci Rep. 2020 Sep 1;10:14351. doi: 10.1038/s41598-020-71264-8 (PMC7462861; doi:10.1038/s41598-020-71264-8)
Supplement: Supplementary file 1 — Supplementary file1 [file 41598_2020_71264_MOESM1_ESM.docx]

**TITLE:** Myocardial proteomic profile in pulmonary arterial hypertension

**AUTHORS:** Mateusz K. Hołda MD, PhD, Aneta Stachowicz PhD, Maciej Suski PhD, Dorota Wojtysiak PhD, Natalia Sowińska DVM, PhD, Zbigniew Arent DVM, PhD, Natalia Palka MD, Piotr Podolec MD, PhD, Grzegorz Kopeć MD, PhD

**Supplementary Table 1 – Echocardiographic parameters measured during the whole experiment (mean ± SD).**

| **Parameter** | **Group** | **Euthanasia day** | **Day 0** | **Day +5** | **Day +10** | **Day +15** | **Day +20** | **Day +24** | **Day +27** | **Day +30** | **Day +33** | **Day +36** | **Day +39** | **Day +42** | **Day +45** | **Day +48** | **Day +51** |
| --- | --- | --- | --- | --- | --- | --- | --- | --- | --- | --- | --- | --- | --- | --- | --- | --- | --- |
| **RVFWTd (mm)** | **early PAH rats** | 0.77 ± 0.04 | 0.52 ± 0.08 | 0.51 ± 0.09 | 0.58 ± 0.07 | 0.66 ± 0.11 | 0.83 ± 0.04 | - | - | - | - | - | - | - | - | - | - |
|  | **non-PAH matched control rats** | 0.57 ± 0.08 | 0.51 ± 0.06 | 0.53 ± 0.05 | 0.56 ± 0.07 | 0.57 ± 0.10 | 0.58 ± 0.09 | - | - | - | - | - | - | - | - | - | - |
|  | **end-stage PAH rats** | 1.03 ± 0.09 | 0.53 ± 0.07 | 0.53 ± 0.06 | 0.57 ± 0.07 | 0.65 ± 0.09 | 0.81 ± 0.06 | 0.87 ± 0.07 | 0.91 ± 0.15 | 0.97 ± 0.10 | 0.95 ± 0.09 | 0.99 ± 0.12 | 1.00 ± 0.09 | 0.98 ± 0.10 | 1.06 ± 0.11 | 1.10 ± 0.06 | 1.18 ± 0.08 |
|  | **non-PAH matched control rats** | 0.66 ± 0.03 | 0.52 ± 0.05 | 0.54 ± 0.08 | 0.53 ± 0.06 | 0.58 ± 0.07 | 0.62 ± 0.06 | 0.60 ± 0.05 | 0.63 ± 0.06 | 0.62 ± 0.06 | 0.65 ± 0.05 | 0.65 ± 0.03 | 0.62 ± 0.05 | 0.67 ± 0.02 | 0.68 ± 0.01 | 0.66 ± 0.03 | 0.67 ± 0.02 |
| **TAPSE (mm)** | **early PAH rats** | 1.02 ± 0.13 | 1.54 ± 0.40 | 1.46 ± 0.33 | 1.32 ± 0.28 | 1.05 ± 0.20 | 0.97 ± 0.10 | - | - | - | - | - | - | - | - | - | - |
|  | **non-PAH matched control rats** | 1.43 ± 0.57 | 1.52 ± 0.46 | 1.48 ± 0.43 | 1.46 ± 0.50 | 1.53 ± 0.48 | 1.42 ± 0.53 | - | - | - | - | - | - | - | - | - | - |
|  | **end-stage PAH rats** | 0.76 ± 0.13 | 1.47 ± 0.52 | 1.37 ± 0.49 | 1.31 ± 0.50 | 1.27 ± 0.57 | 1.10 ± 0.45 | 1.03 ± 0.39 | 0.90 ± 0.34 | 0.85 ± 0.31 | 0.83 ± 0.26 | 0.78 ± 0.20 | 0.78 ± 0.23 | 0.72 ± 0.20 | 0.70 ± 0.13 | 0.68 ± 0.16 | 0.67 ± 0.09 |
|  | **non-PAH matched control rats** | 1.21 ± 0.40 | 1.44 ± 0.53 | 1.41 ± 0.51 | 1.38 ± 0.43 | 1.43 ± 0.56 | 1.36 ± 0.43 | 1.28 ± 0.37 | 1.26 ± 0.29 | 1.23 ± 0.36 | 1.20 ± 0.38 | 1.21 ± 0.35 | 1.23 ± 0.33 | 1.21 ± 0.24 | 1.20 ± 0.21 | 1.19 ± 0.20 | 1.23 ± 0.23 |
| **PAAT/CL** | **early PAH rats** | 0.21 ± 0.06 | 0.23 ± 0.06 | 0.21 ± 0.05 | 0.22 ± 0.05 | 0.20 ± 0.05 | 0.23 ± 0.06 | - | - | - | - | - | - | - | - | - | - |
|  | **non-PAH matched control rats** | 0.23 ± 0.07 | 0.24 ±  0.03 | 0.25 ± 0.06 | 0.23 ± 0.03 | 0.21 ± 0.05 | 0.25 ± 0.07 | - | - | - | - | - | - | - | - | - | - |
|  | **end-stage PAH rats** | 0.15 ± 0.06 | 0.23 ±  0.04 | 0.26 ±  0.05 | 0.23 ±  0.06 | 0.22 ±  0.04 | 0.23 ±  0.03 | 0.22 ±  0.02 | 0.20 ±  0.03 | 0.19 ±  0.04 | 0.17 ±  0.06 | 0.16 ±  0.03 | 0.14 ±  0.01 | 0.15 ±  0.02 | 0.13 ±  0.03 | 0.14 ±  0.01 | 0.15 ±  0.02 |
|  | **non-PAH matched control rats** | 0.22 ± 0.05 | 0.23 ± 0.04 | 0.23 ± 0.04 | 0.21 ± 0.05 | 0.22 ± 0.04 | 0.20 ± 0.06 | 0.22 ± 0.04 | 0.22 ± 0.05 | 0.21 ± 0.04 | 0.19 ± 0.07 | 0.22 ± 0.04 | 0.22 ± 0.05 | 0.24 ± 0.07 | 0.21 ± 0.05 | 0.20 ± 0.06 | 0.22 ± 0.03 |

PAAT/CL – pulmonary artery acceleration time normalized to cycle length, PAH – pulmonary arterial hypertension, RVFWTd – end-diastolic right ventricular free wall thickness, TAPSE – tricuspid annular plane systolic excursion

**Supplementary Table 2 – Abundance of left and right ventricle myocardium proteins that are significantly altered in both early PAH and end-stage PAH.**

| **Sample** | **protein name** | **early PAH  fold change** | **end-stage PAH  fold change** |
| --- | --- | --- | --- |
| **Left ventricle myocardium** | Serine protease inhibitor A3K | 1.16 | -1.15 |
|  | Myosin-7 | 1.12 | 1.16 |
| **Right ventricle myocardium** | Myosin-7 | 1.17 | 1.40 |
|  | Acetyl-CoA acetyltransferase, mit. | 1.14 | -1.14 |
|  | Methylmalonate-semialdehyde dehydrogenase [acylating], mit. | 1.12 | -1.12 |
|  | Long-chain specific acyl-CoA dehydrogenase, mit. | 1.09 | -1.11 |
|  | L-lactate dehydrogenase A chain | -1.12 | 1.12 |

PAH – pulmonary arterial hypertension
